# Ecological Risk of Heavy Metals and a Metalloid in Agricultural Soils in Tarkwa, Ghana

**DOI:** 10.3390/ijerph120911448

**Published:** 2015-09-11

**Authors:** Nesta Bortey-Sam, Shouta M. M. Nakayama, Osei Akoto, Yoshinori Ikenaka, Elvis Baidoo, Hazuki Mizukawa, Mayumi Ishizuka

**Affiliations:** 1 Laboratory of Toxicology, Department of Environmental Veterinary Sciences, Graduate School of Veterinary Medicine, Hokkaido University, Kita 18, Nishi 9, Kita ku, Sapporo 060–0818, Japan; E-Mails: borteysam@yahoo.com (N.B.-S.); shouta-nakayama@vetmed.hokudai.ac.jp (S.M.M.N.); y_ikenaka@vetmed.hokudai.ac.jp (Y.I.); 2 Department of Chemistry, Kwame Nkrumah University of Science and Technology, Private Mail Bag, KNUST, Kumasi, Ghana; E-Mails: wofakmann@yahoo.com (O.A.); elvixbaid@yahoo.com (E.B.); 3 Department of Environmental Veterinary Sciences, Graduate School of Veterinary Medicine, Hokkaido University, Kita 18, Nishi 9, Kita ku, Sapporo 060–0818, Japan; E-Mail: hazuki.mizukawa@vetmed.hokudai.ac.jp

**Keywords:** heavy metals, metalloid, agricultural soil, Tarkwa, integrated pollution, ecological risk

## Abstract

Heavy metals and a metalloid in agricultural soils in 19 communities in Tarkwa were analyzed to assess the potential ecological risk. A total of 147 soil samples were collected in June, 2012 and analyzed for As, Cd, Co, Cr, Cu, Hg, Ni, Pb and Zn. Mean concentrations (mg/kg dw) of heavy metals in the communities decreased in order of Zn (39) ˃ Cr (21) ˃ Pb (7.2) ˃ Cu (6.2) ˃ As (4.4) ˃ Ni (3.7) ˃ Co (1.8) ˃ Hg (0.32) ˃ Cd (0.050). Correlations among heavy metals and soil properties indicated that soil organic matter could have substantial influence on the total contents of these metals in soil. From the results, integrated pollution (C_deg_) in some communities such as, Wangarakrom (11), Badukrom (13) and T–Tamso (17) indicated high pollution with toxic metals, especially from As and Hg. Potential ecological risk (RI) indices indicated low (Mile 7) to high risks (Wangarakrom; Badukrom) of metals. Based on pollution coefficient (*C^i^_f_*), C_deg_, monomial ecological risk (*E^i^_r_*) and RI, the investigated soils fall within low to high contamination and risk of heavy metals to the ecological system especially plants, soil invertebrates and/or mammalian wildlife. This represented moderate potential ecological risk in the study area, and mining activities have played a significant role.

## 1. Introduction

Heavy metal and metalloids pollution in the environment has become an important issue worldwide due to the abundance of sources, their environmental persistence, and potential toxicity to ecological receptors [1,2,3,4,5,6]. The accumulation of heavy metals in soils is affected by many environmental variables, including parent material, soil properties, as well as by human activities and point sources. The rapid development and industrialization including mining activities over recent decades in Ghana’s Tarkwa region has brought significant environmental problems. For example, a number of researchers have documented widespread contamination in this region with toxic metals, such as arsenic and mercury, in water, soil, plant, food and humans [7,8,9,10,11].

Many metals bioaccumulate in the edible parts of crops and thus negatively impact the health of human, animals and the ecosystem [12,13]. Amongst the many potential metals that may contaminate agricultural and ecological systems, arsenic (As), cadmium (Cd), cobalt (Co), chromium (Cr), copper (Cu), mercury (Hg), nickel (Ni), lead (Pb) and zinc (Zn) are perhaps the most important [14]. Many of these elements, especially As and Hg, are found in rather high concentrations in mining areas of Ghana [7,8,9,11,15].

Studies conducted by Bortey-Sam *et al.* [11] and Hayford *et al.* [15] on the impact of gold mining in soil and foods collected around mining communities in Tarkwa showed high levels of some toxic metals including As and Hg. Similarly, work done by Asante *et al.* [8] showed high concentrations of As and manganese (Mn) in borehole, well and river/stream water in Tarkwa. Despite the wide and numerous studies of toxic metals concentrations in various environmental and biological samples in Ghana, there is limited or no data from literature on the potential ecological risk of heavy metals and a metalloid in agricultural soils in Tarkwa, Ghana. The objectives of this study were therefore to increase understanding of the ecological risk that may be posed by metal contamination in the Tarkwa region of Ghana. Specifically, the study aimed to determine the concentrations of heavy metals and a metalloid in agricultural soils in 19 communities in Tarkwa; to identify the relationship between heavy metals and soil properties; to identify the potential sources of the metals; and to estimate the potential risk of heavy metals and a metalloid in agricultural soils to the ecological system in Tarkwa.

## 2. Materials and Methods

### 2.1. Study Area

Tarkwa (05°18′00″N; 01°59′00″W) is a town in the southwest of Ghana, located about 120 miles west of the capital city, Accra. As of 2010, Tarkwa was estimated to have a population of 90,477 [16]. It is a noted centre for gold and Mn mining. Tarkwa mine, which is a large open–cast gold mine, is situated to the northwest of the town, and Nsuta manganese mine is situated to the east. Tarkwa has a long history of gold mining and perhaps the greatest concentration of mining companies including illegal mining (galamsey) activities in the West African sub-region [17].

### 2.2. Sample Collection and Analysis

**Figure 1:**
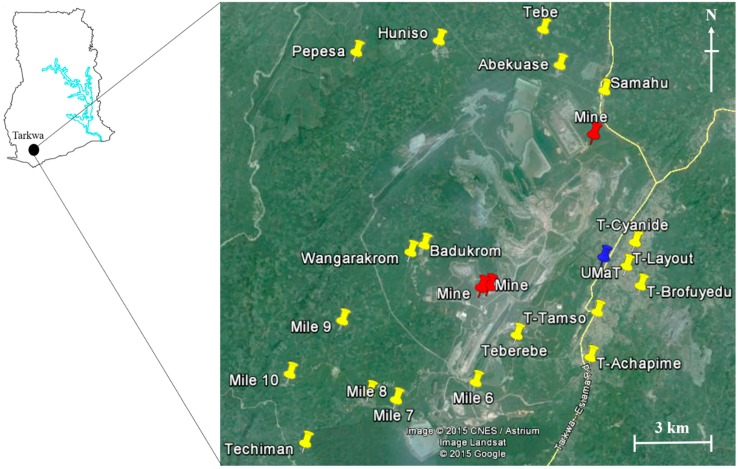
Map showing surface soil sampling locations in Tarkwa, Ghana (yellow, red and blue pins indicate sampled communities, gold mines and reference site (UMaT), respectively).

In June 2012, a total of 142 soil samples (0–10 cm top layer) were randomly collected from 19 communities in Tarkwa. These sites were selected because of the agricultural activities and also to represent a wide area of the town. Global positioning system (GPS) was used to locate the sampling locations/positions and some sites in Badukrom, Wangarakrom and T-Tamso were approximately 3, 3.4 and 5.2 km away from the mines, respectively. Communities such as Pepesa, Mile 10 and Techiman were farthest away from the mines with average distances of 11, 13 and 14 km, respectively (Figure 1).

In addition, due to the lack of background concentrations in agricultural soils in Tarkwa, Ghana, five soil samples were collected from the University of Mines and Technology (UMaT) campus for data comparison (reference values), and to evaluate the extent of metal pollution in this study. UMaT is a public university located in Tarkwa, Ghana, and because of the low vehicular movement and industrial (mining) activities, heavy metals and metalloids from point sources were assumed to be negligible. Samples were collected using a stainless steel scoop and stored in labeled corning tubes (Corning Incorporated, New York, USA) [18]. The soil samples obtained were stored at −20 °C in the Department of Chemistry, KNUST, Ghana and later transported to the Laboratory of Toxicology, Graduate School of Veterinary Medicine, Hokkaido University, Japan, where they were stored at −30 °C until analysis. A map showing the sampling locations is presented in Figure 1.

Prior to chemical analyses, the soil samples were air dried at room temperature and passed through a 2 mm sieve to remove coarse debris [18]. Approximately 0.5 g of soil sample was weighed into a prewashed digestion vessel. The samples were digested (Speedwave two, Berghof, Germany) using 10 ml of 60% nitric acid (Kanto Chemical Corporation, Tokyo, Japan). The microwave unit was calibrated to a temperature of 200 °C and digestion was allowed for 45 min at 180 psi. After cooling, samples were filtered into corning tubes (Corning Incorporated, New York, USA) using ashless filter paper 5B (Advantec, Tokyo, Japan). The solution was standardized to 50 ml using distilled, deionised water. Method blanks were prepared using the same procedure.

Concentrations of As, Cd, Co, Cr, Cu, Ni, Pb and Zn were measured by an Inductively Coupled Plasma–Mass Spectrometer (ICP–MS; 7700 series, Agilent technologies, Tokyo, Japan) and expressed in mg/kg dry weight (dw). On the other hand, concentration of total Hg (Hg) in soil sample was measured by thermal decomposition, gold amalgamation and atomic absorption spectrophotometry (Mercury Analyzer, MA–3000, Nippon Instruments Corporation, Tokyo, Japan), after preparation of the calibration standard.

### 2.3. Quality Control and Quality Assurance

For quality control, blanks were analyzed after every 10 sample analyses. The instrument was calibrated using standard solutions of the respective metals (to establish standard curves before metal analysis). All chemicals and standard stock solutions were of analytical–reagent grade (Wako Pure Chemicals, Osaka, Japan). The detection limits (ng/g) of As, Cd, Co, Cr, Cu, Ni, Pb, and Zn were 0.002, 0.001, 0.0001, 0.007, 0.004, 0.004, 0.001, 0.046, respectively. For metals, reference materials SRM 1944 (New York/ New Jersey Waterway Sediment) and BCR–320 (Channel Sediment, IRMM, Belgium) were used for method validation. Replicate analyses of these reference materials showed good accuracy with recovery rates ranging from 80%–115%. Recovery rates (%) of Hg for the three certified reference materials (BCR–320R, SRM 1944, and DOLT–4) ranged from 92–103. The detection limit of Hg in soil samples was 2.0 pg total Hg.

The water content of each soil sample was measured after 12 h of drying in an oven at 105 °C. Soil organic matter (SOM) content was determined by loss of weight on ignition at oven temperature of 600 °C for 5 h. pH was measured in a soil deionized water suspension (soil: water, 1:2.5 by volume) by a calibrated pH meter.

### 2.4. Statistical Analysis

Statistical analyses were performed using SPSS 20.0 (IBM SPSS Inc., Chicago, USA). Kolmogorov-Smirnov and Shapiro-Wilk’s tests were used to determine the normality of data and was considered statistically significant if *p* value was less than 0.05. Statistical analyses were carried out after data were log transformed (normalized). Principal component analysis (PCA) is a statistical method used to determine components that are linear combinations of the original variables and was performed using JMP 10 statistical software (SAS Institute). In order to identify the important parameters which affect the chemistry of soil and to investigate the possible sources of different metals, Pearson’s correlation matrix and PCA were used, respectively. The principal components based on log transformed data were extracted with eigenvalues >1 through a varimax rotation. Spearman and Pearson’s correlation were used to determine the relationship between concentrations of metals and distance from the mines, and was considered significant if *p* value was less than 0.05.

## 3. Results and Discussion

### 3.1. Concentrations of Heavy Metals and a Metalloid in Soils

Table 1 shows the mean (±SD) concentrations of As, Cd, Co, Cr, Cu, Hg, Ni, Pb, and Zn in soil in 19 communities in Tarkwa. From Table 1, the mean concentrations of eight heavy metals and a metalloid decreased in order of Zn ˃ Cr ˃ Pb ˃ Cu ˃ As ˃ Ni ˃ Co ˃ Hg ˃ Cd. The high variability in concentration was illustrated by the Shapiro-Wilks and Kolmogorov-Smirnov (K-S) tests, showing an abnormal distribution of raw data for all the heavy metals (Table 1; *p* < 0.0001). This variability could be due to the large sampling area of 19 communities. Industrial activities including mining could be associated with heavy metals discharge in some areas and could explain this variability.

The average concentrations of metals in the 19 communities were generally below the corresponding ecological-soil screening levels (ECO-SSL) for plants, soil invertebrates and mammalian wildlife established by the United States Environmental Protection Agency, USEPA [19,20] (Table 1). However, some communities/sample sites showed higher values than the USEPA ECO-SSL [19,20] and Kabata-Pendias and Sadurski [21] recommended levels (Table 1). For instance, two sites in Badukrom and Wangarakrom had higher Hg concentrations than the Maximum Allowable Concentrations (MAC) of 0.5–5 mg/kg in agricultural soils [21]. These results indicated a possible influence of artisanal and small-scale gold mining activities in the study area since Hg is used to amalgamate gold from ore. Further, such influence is consistent with the high coefficients of variation (CV) found for most of the measured heavy metals, (CV values ranged from 50% [Cu]–147% [Ni]; Table 1) [22,23]. As shown in Table 1, the highest mean concentrations of Pb and Hg were in T–Layout and Badukrom, respectively, while highest mean concentrations of As, Cd, Co, Cr, Cu, Ni and Zn were in T–Tamso. The high levels of metals and a metalloid in soil in T-Tamso could be attributed to the proximity of some sample sites to the mines.

**Table 1:** WC%, SOM%, soil pH and mean (±SD) concentrations (mg/kg dw)) of heavy metals and a metalloid in soils in Tarkwa.

Sample Sites	*n*		WC	SOM	Soil pH	As	Cd	Co	Cr	Cu	Hg	Ni	Pb	Zn
Teberebe	8	Mean	1.1	2.5	7.6	2.6	0.038	3.0	35	8.9	0.072	4.5	6.1	39
		SD	0.48	1.5	0.11	1.2	0.017	2.4	20	6.5	0.044	2.9	1.7	17
Mile 6	7	Mean	0.94	2.2	7.5	2.2	0.020	2.1	30	9.3	0.018	2.6	3.2	12
		SD	0.65	1.9	1.1	1.2	0.010	1.1	16	6.8	0.018	1.2	1.7	11
Mile 7	8	Mean	0.91	1.8	7.3	1.0	0.011	0.67	13	2.8	0.030	1.1	1.5	9.7
		SD	0.31	1.1	1.1	0.51	0.010	0.45	17	1.4	0.011	0.64	0.81	3.3
Mile 8	7	Mean	0.88	1.8	7.1	1.3	0.022	0.74	9.9	2.8	0.19	1.5	2.3	23
		SD	0.53	0.72	1.0	0.72	0.011	0.68	4.2	1.6	0.26	0.91	0.75	13
Techiman	8	Mean	0.66	1.8	6.2	1.0	0.020	1.6	11	5.5	0.17	3.1	2.7	32
		SD	0.78	1.0	1.2	0.44	0.021	1.7	5.0	4.0	0.14	3.0	1.6	45
Mile 9	8	Mean	0.50	2.1	5.8	1.8	0.011	0.80	15	2.5	0.033	1.6	2.0	11
		SD	0.19	2.0	0.31	1.5	0.010	0.64	15	1.9	0.025	1.1	1.0	7.8
Mile 10	8	Mean	0.44	1.3	7.1	0.96	0.020	0.72	27	2.2	0.13	1.0	2.1	29
		SD	0.19	0.34	0.051	0.43	0.016	0.53	34	1.0	0.090	0.21	0.23	25
Wangarakrom	8	Mean	0.66	2.8	6.9	5.4	0.021	1.4	15	3.7	1.9	3.2	2.5	19
		SD	0.33	1.0	0.021	6.3	0.018	1.6	5.6	2.2	1.1	3.0	0.90	11
Badukrom	8	Mean	2.5	2.0	6.9	12	0.013	0.37	9.6	5.9	2.4	1.3	2.4	27
		SD	3.7	1.2	0.030	10	0.010	0.12	4.9	8.2	1.7	0.57	0.98	30
Samahu	7	Mean	2.1	2.4	7.1	4.5	0.030	1.2	38	5.8	0.11	2.4	8.3	36
		SD	2.3	1.7	0.049	4.3	0.026	0.84	39	5.0	0.12	1.8	11	29
Abekuase	9	Mean	2.1	2.5	7.0	3.2	0.024	1.2	9.2	3.8	0.050	1.8	3.8	38
		SD	1.1	1.1	0.084	1.0	0.023	1.2	4.8	4.2	0.021	1.2	2.7	47
Tebe	9	Mean	1.9	2.9	6.9	2.8	0.011	1.8	12	5.6	0.051	2.1	4.1	18
		SD	1.2	1.7	0.074	1.4	0.010	3.2	9.3	5.5	0.035	1.9	2.0	18
Huniso	7	Mean	0.99	1.5	7.2	1.5	0.052	0.76	8.0	4.1	0.13	1.3	13	86
		SD	0.85	0.84	0.11	0.79	0.034	0.32	3.7	2.8	0.12	0.53	15	69
Pepesa	10	Mean	1.9	1.9	7.3	4.9	0.042	0.89	12	6.9	0.20	1.9	5.5	78
		SD	0.79	0.81	0.10	8.1	0.034	0.69	4.2	7.6	0.24	1.2	3.3	73
T-Cyanide	7	Mean	1.2	2.4	7.5	2.7	0.081	1.4	23	8.6	0.18	3.2	16	49
		SD	0.75	0.24	0.087	1.2	0.014	0.81	16	5.0	0.036	1.3	4.6	21
T–Layout	6	Mean	1.4	1.1	7.4	2.7	0.11	1.4	16	7.1	0.11	3.0	27	78
		SD	1.1	0.78	0.062	1.4	0.14	0.70	10	7.4	0.10	1.7	37	108
T–Brofuyedu	5	Mean	0.95	1.9	7.3	8.6	0.058	1.3	18	7.7	0.061	2.9	6.0	32
		SD	0.03	1.8	0.021	10	0.027	1.4	14	7.2	0.034	2.7	2.2	11
T–Achapime	6	Mean	1.5	1.9	7.2	1.4	0.046	0.66	12	4.9	0.73	1.9	6.4	45
		SD	0.94	0.37	0.056	0.13	0.0012	0.04	0.01	0.63	0.59	0.17	0.68	1.8
T–Tamso	6	Mean	1.2	3.2	7.5	27	0.43	9.2	77	16	0.42	28	14	118
		SD	0.42	1.3	0.23	13	0.20	4.4	46	9.2	0.014	14	3.8	85
Minimum			0.44	1.1	5.8	0.96	0.010	0.37	8.0	2.2	0.018	1.0	1.5	9.7
Maximum			2.5	3.7	7.6	27	0.43	9.2	77	16	2.4	28	27	118
Median			1.1	2.2	7.3	2.7	0.024	1.3	15	5.8	0.11	2.5	5.5	32
Average			1.3	2.4	7.2	4.4	0.052	1.8	21	6.2	0.32	3.7	7.2	39
SD			0.61	0.69	0.42	5.1	0.067	1.8	15	3.1	0.36	5.5	6.2	27
CV			47	29	5.8	116	111	101	73	50	113	147	85	70
Tarkwa ( *n* = 142)			0.0–11	0.0–10	4.3–8.8	0.3–37	nd–0.58	0.11–14	2.0–199	0.5–44	nd–6.7	0.3–38	0.4–78	1.1–232
Skewness			3.4	1.3	–1.6	4.1	5.6	2.4	4.00	2.3	8.0	4.1	4.3	2.6
Kurtosis			16	2.4	6.8	19	37	6.8	19	9.3	67	21	21	7.8
K–S/Shapiro–Wilk *p*			˂0.0001	˂0.0001	˂0.0001	˂0.0001	˂0.0001	˂0.0001	˂0.0001	˂0.0001	˂0.0001	˂0.0001	˂0.0001	˂0.0001
Reference values ^#^			1.2	6.8	7.3	5.8	0.39	3.6	33.18	21	0.24	6.7	52	72
USEPA ^b^						18–46	0.36–140	13–230	–	49–80	0.5-5 *	38–280	56–120	79–160
World range ^c^						1.0–15	0.07–1.1	0.1–20	5–120	6.0–60		1–200	10.0–70	17–125

Notes: *n*: number of samples; * Indicates Maximum Allowable Concentration (MAC) of Hg in agricultural soils by Kabata-Pendias and Sadurski [21]; **^b^** indicates USEPA Ecological-Soil Screening Levels for plants, soil invertebrates and mammalian wildlife [19,20]; **^c^** indicates recommended levels of heavy metals in soil by Kabata–Pendias and Pendias [24]; **^#^** indicates reference values (UMaT). Bold values indicates higher concentrations than, USEPA [19,20] and Kabata–Pendias and Pendias [24]; nd: not detected.

### 3.2. Correlation between Heavy Metals and Soil Properties

Correlation between heavy metals and selected soil properties was analyzed by Pearson’s correlation matrix (Table 2). Soil properties play an important role in the mobility and bioavailability of heavy metals, thus influencing their distribution in soils [12,25]. This role is generally illustrated by good correlations between heavy metal concentrations and pH, as well as SOM [26,27]. However, only weak correlations were found in some studies [22]. In the present study, significant correlations were observed between As, Cd, Co, Cr, Cu, Ni, Pb and SOM (r = 0.18–0.51, *p* < 0.0001–0.05), indicating that SOM has substantial influence on the total contents of these metals in soil. Similar result was reported by Gjoka *et al.* [26]. However, no significant correlations were found between pH and heavy metals, which is similar to the results by Manta *et al.* [22] and Al–Khashman and Shawabkeh [28].

Lack of significant correlation between soil properties and heavy metals could be attributed to a continuous input [27,29] since the release and transport of heavy metals are complex processes [30]. Another possible explanation could be variations in soil type, fertilizer use, and cultivation system within the sampling area [27,30]. No significant correlation was found between Hg and the other metals (*p* ˃ 0.05) except for As, indicating a specific source for As and Hg. The sources for Hg could be geochemical and/or anthropogenic [31], since it is used in the amalgamation of gold [32]. On the other hand, significant positive relationships (*p* < 0.0001) were observed between As and Cd (r = 0.48), Cd and Zn (r = 0.62), Cd and Pb (r = 0.44), Ni and Cr (r = 0.57), and Pb and Zn (r = 0.39). In addition, significantly weak relationships (*p* < 0.05 or 0.01) were found between Zn and As (r = 0.23), and Zn and Ni (r = 0.25) (Table 2). The significantly positive correlations among these elements suggested, to some extent, a common source [28].

### 3.3. Sources of Metals in Soil Identified by PCA

**Figure 2:**
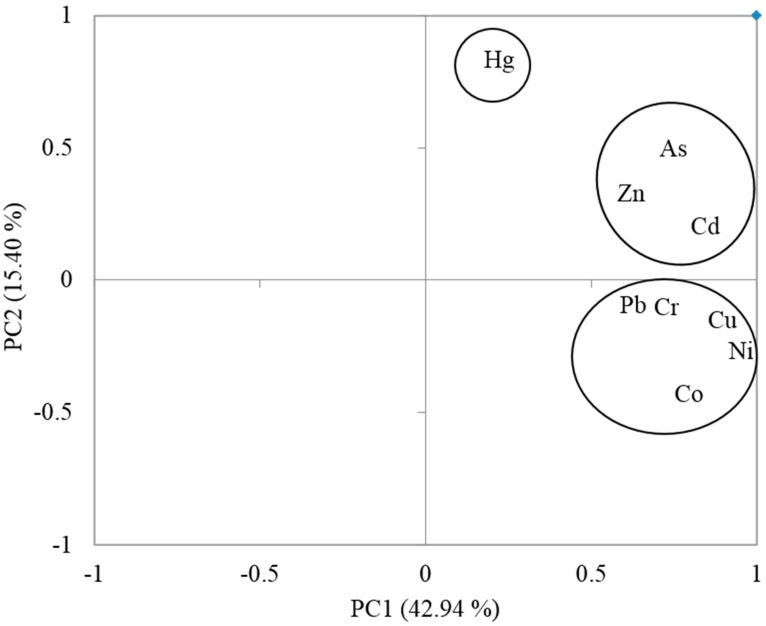
Distribution pattern of metals in agricultural soils in Tarkwa characterized by PCA.

In this study, three principal components (PC1, PC2, and PC3) were extracted (with eigenvalues >1) accounting for 72.8% of the total variances. As shown in Figure 2, PC1, the most important component, explained 42.9% of the total variance and was characterized by high loadings of Co, Cu, Cr, Ni, and Pb. The input of these metals could mainly result from atmospheric deposition, as a consequence of an increase in industrial activities such as mining and smelting processes [33,34,35]. The concentrations of Cr, Co and Pb in the study area could also be attributed to the weathering of the Tarkwanian rock system. The Tarkwanian rock system contains high concentration of Cr, Co and Pb. Other sources of Cr, Co and Pb in the study area is the occasional discharge of acid industrial wastes or mine drainage which increases Cr, Co and Pb levels in surface soils in the study area [9].

**Table 2:** Pearson’s correlation matrix of heavy metals and soils properties in Tarkwa.

Metals/Soil Properties	WC%	SOM%	Soil pH	As	Cd	Co	Cr	Cu	Hg	Ni	Pb	Zn
WC%	1											
SOM%	0.33	1										
soil pH	0.04	−0.06	1									
As	0.03	0.27 **	−0.01	1								
Cd	−0.02	0.18 *	0.09	0.48 ***	1							
Co	0.06	0.40 ***	0.11	0.27 **	0.35 ***	1						
Cr	0.08	0.51 ***	0.03	0.54 ***	0.27 ***	0.31 ***	1					
Cu	0.02	0.47 ***	0.11	0.37 ***	0.41 ***	0.59 ***	0.50 ***	1				
Hg	−0.07	−0.05	−0.06	0.45 ***	0.06	−0.09	−0.03	0.08	1			
Ni	0.01	0.49 ***	0.06	0.50 ***	0.56 ***	0.76 ***	0.57 ***	0.68 ***	−0.03	1		
Pb	0.04	0.34 ***	0.05	0.09	0.44 ***	0.28 **	0.16 *	0.37 ***	−0.02	0.36 ***	1	
Zn	−0.00	0.07	0.03	0.23 *	0.62 ***	0.13	0.10	0.34 ***	0.12	0.25 ***	0.39 ***	1

Notes: * Indicates *p* ˂ 0.05; ** Indicates *p* ˂ 0.001; *** Indicates *p* ˂ 0.0001.

PC2 explained 15.4% of the total variances (Figure 2) and was dominated by high loadings of As, Cd, and Zn. Similar to PC1, PC2 also represents anthropogenic contamination, probably resulting from irrigation with sewage water [36,37] and the use, and sometimes abuse, of phosphate fertilizers and organic manures [23,29,38]. Huge amount of phosphate [39] causes considerable additions of As and Cd. The use of livestock manure adds As and Cd to agricultural soils [40]. In addition, blasting of the gold bearing rock is the most common method of obtaining the ore. The miners engage in surface and subsurface mining [41,42]. The levels of As in the soils could also be due to the nature of the gold bearing ore, which is mineralized pyrites and arsenopyrates. Processing of the ore involves roasting and this results in the production of arsenic trioxide gas which is distributed throughout the study area by air current. As is toxic and due to its non-biodegradable nature, it could accumulate in surface soil and water [32].

Cd is soft, ductile and is obtained as a by-product from the smelting of Zn ores. It is also found in chalophile as a mineral called greenockite, CdS. Cd in soils from the study area may come from the mining and processing of Zn and chalophilic metals [9]. The presence of Zn in the environment is associated with mining and smelting, which pollutes the air, water and soil, and ultimately undergoes oxidation to release Zn^2+^ ions [9]. Thus, PC2 could be regarded as representing mainly the contribution of mining and use of fertilizers/manure. This is in agreement with a study by Asante *et al.*, [8] which indicated that there could be other sources of As contamination in Tarkwa other than mining activities.

PC3 explained 14.5% of the total variances and was totally dominated by high loading of Hg. The levels of Hg in soils from some sites could be problematic, as concentrations exceeded the maximum values permitted in agricultural soils [21]. In Ghana, amalgamation using Hg (popularly known as "galamsey"), is the preferred gold recovery method employed by almost all artisanal gold miners because it is a very simple, inexpensive and an easier to use technique [32]. The high levels of Hg in soils could therefore be due to contamination from the mining processes.

### 3.4. Assessment of Potential Ecological Risk

The potential ecological risk (RI) is a commonly used indicator to express a comprehensive assessment of the harmful effects of heavy metals and a metalloid in the environment, including soils and sediments. The RI was calculated using the following equations [43,44]:

(1) $${C^{i}}_{f}\;=C^{i}/{C^{i}}_{n}$$

(2) $$C_{deg}=\;\sum {C^{i}}_{f}$$

(3) $${E^{i}}_{r}=\;{T^{i}}_{r}\times \;{C^{i}}_{f}$$

(4) $$\mathrm{RI}\;=\;\sum {E^{i}}_{r}$$

Where *C^i^_f_* is the pollution coefficient of a metal which can reflect the pollution character of the investigated region but cannot reveal the ecological effects. *C^i^* is the measured values of heavy metals in surface soils. *C^i^_n_* is the reference values of the heavy metals in soil/sediments. The concentrations of metals (mg/kg dw) in soil samples collected from UMaT were used as reference (Table 1). The *C^i^_f_* of each metal was calculated and classified as either low (*C^i^_f_ ≤* 1), middle (1 < *C^i^_f_ ≤* 3) or high (*C^i^_f_* > 3) [45].

C_deg_ represents the integrated pollution level in the environment, and is expressed as the sum of *C^i^_f_* for all examined metals. The four pollution levels may be distinguished as: C_deg_ < 5, low pollution; 5 ≤ C_deg_ < 10, medium pollution; 10 ≤ C_deg_ < 20, high pollution; and C_deg_ ≥ 20, very high pollution [46]. *E^i^_r_* is the monomial potential ecological risk factor of the individual heavy metal and *T^i^_r_* is the metal toxic factor (based on the standardized heavy metal toxic factor). Referring to Hakanson [43], we used the following *T^i^_r_* values: Hg = 40; Cd = 30; As = 10; Cu = Pb = Ni = 5, Cr = 2, and Zn = 1. RI is defined as the sum of *E^i^_r_* for all heavy metals and has been grouped into four categories by Zhu *et al.* [44] as shown in Table 3.

**Table 3:** Categories of *E^i^_r_* and RI [43,44].

*E^i^_r_*	Ecological Risk Level of Single Factor Pollution	RI Value	General Level of Potential Ecological Risk
*E^i^_r_* ˂ 40	Low risk	RI ≤ 50	Low risk
40 ≤ *E^i^_r_* ˂ 80	Moderate risk	50 ˂ RI ≤100	Moderate risk
80 ≤ *E^i^_r_* ˂ 160	Considerable risk	100 ˂ RI ≤ 200	Considerable risk
*E^i^_r_* ˂ 320	High risk	RI ˃ 200	High risk
*E^i^_r_* ≥ 320	Very high risk		

The *C^i^_f_* values for the measured heavy metals and metalloid ranged from As (0.16–4.6), Cd (0.026–1.1), Cr (0.24–2.3), Cu (0.11–0.80), Hg (0.080–10), Ni (0.15–4.2), Pb (0.030–0.53) and Zn (0.13–1.6). This suggested a low to high pollution level (Table 4; [45]). The range of C_deg_ was 1.2–17, with an average of 4.5. From Table 4, average C_deg_ (4.5) indicated low pollution for most soil samples (75%). However, the C_deg_ for Wangarakrom (11), Badukrom (13) and T-Tamso (17) indicated high pollution of toxic metals, especially from As and Hg (Table 4).

**Table 4:** *C^i^_f_* and C_deg_ of heavy metals and a metalloid in surface soils in Tarkwa.

Sample Sites	As	Cd	Cr	Cu	Hg	Ni	Pb	Zn	C_deg_
Teberebe	0.46	0.10	**1.0**	0.42	0.30	0.67	0.12	0.54	3.6
Mile 6	0.39	0.044	0.91	0.44	0.08	0.40	0.063	0.18	2.5
Mile 7	0.18	0.030	0.42	0.13	0.11	0.16	0.030	0.13	1.2
Mile 8	0.22	0.060	0.30	0.13	0.78	0.23	0.044	0.33	2.0
Techiman	0.18	0.052	0.34	0.26	0.70	0.46	0.053	0.45	2.4
Mile 9	0.32	0.030	0.46	0.12	0.14	0.24	0.040	0.16	1.5
Mile 10	0.16	0.051	0.84	0.11	0.55	0.15	0.041	0.40	2.3
Wangarakrom	0.93	0.055	0.47	0.17	**8.1**	0.48	0.051	0.26	**11**
Badukrom	**2.2**	0.041	0.29	0.28	**10**	0.20	0.050	0.38	**13**
Samahu	0.79	0.064	**1.1**	0.28	0.44	0.36	0.16	0.50	3.7
Abekuase	0.55	0.062	0.28	0.18	0.19	0.27	0.074	0.53	2.1
Tebe	0.49	0.026	0.38	0.27	0.19	0.32	0.080	0.25	2.0
Huniso	0.26	0.13	0.24	0.20	0.56	0.20	0.26	**1.2**	3.0
Pepesa	0.84	0.11	0.37	0.33	0.84	0.28	0.11	**1.0**	3.9
T–Cyanide	0.47	0.21	0.72	0.41	0.73	0.47	0.31	0.69	4.0
T–Layout	0.47	0.28	0.49	0.34	0.48	0.46	0.53	**1.08**	4.1
T–Brofuyedu	**1.4**	0.15	0.56	0.36	0.25	0.43	0.12	0.45	3.8
T–Achapime	0.25	0.12	0.36	0.23	**3.0**	0.28	0.12	0.62	**5.0**
T–Tamso	**4.6**	**1.1**	**2.3**	0.80	**1.7**	**4.2**	0.27	**1.6**	**17**
Minimum	0.16	0.026	0.24	0.11	0.080	0.15	0.030	0.13	1.2
Maximum	4.6	1.1	2.3	0.80	10	4.2	0.53	1.6	17
Average	0.79	0.14	0.63	0.29	1.4	0.53	0.14	0.57	4.5

Notes: Bold indicates high *C^i^_f_* and C_deg_ values (*i.e.*, middle to high pollution) based on: (**a**) low (*C^i^_f_ ≤* 1), middle (1 < *C^i^_f_ ≤* 3) or high (*C^i^_f_* > 3) [45]; (**b**) C_deg_ < 5, low pollution; 5 ≤ C_deg_ < 10, medium pollution; 10 ≤ C_deg_ < 20, high pollution; and C_deg_ ≥ 20, very high pollution [46].

Hakanson [43] and Zhu *et al.* [44] defined five categories of *E^i^_r_* (Table 3) and four categories of RI. As shown in Table 5, the maximum *E^i^_r_* values for As (46) and Hg (400) were higher than those of the other metals. This result suggested a moderate to very high risk of As (T–Tamso) and Hg (Badukrom), respectively, to the ecological system especially plants, soil invertebrates and/or mammalian wildlife. Similarly the *E^i^_r_* of Hg from T–Achapime (120) and Wangarakrom (324) indicated considerable to high ecological risk (Table 3 and Table 5). The *E^i^_r_* difference between As/Hg and the other metals resulted from their high toxic factors, *T^i^_r_* [31] and high concentration at some sites possibly due to their proximity to the mines (Figure 1) or illegal mining activities. In fact, the CV of Hg from the sampling communities was 113% (Table 1), indicating high Hg concentrations in some communities.

**Table 5:** *E^i^_r_* and RI of heavy metals and a metalloid in surface soils in Tarkwa.

Sample Sites	As	Cd	Cr	Cu	Hg	Ni	Pb	Zn	RI
Teberebe	4.5	2.9	2.1	2.1	11	3.3	0.59	0.54	28
Mile 6	3.8	1.3	1.8	2.2	3.1	1.9	0.31	0.18	14
Mile 7	1.8	0.90	0.84	0.67	4.5	0.81	0.15	0.13	9.9
Mile 8	2.2	1.6	0.60	0.67	31	1.1	0.22	0.33	37
Techiman	1.7	1.5	0.68	1.3	28	2.3	0.27	0.45	36
Mile 9	3.2	0.89	0.92	0.59	5.5	1.2	0.20	0.16	12
Mile 10	1.6	1.5	1.6	0.53	21	0.75	0.21	0.40	28
Wangarakrom	9.3	1.6	0.94	0.87	**324**	2.4	0.25	0.26	**339**
Badukrom	22	1.0	0.58	1.4	**400**	1.0	0.24	0.38	**427**
Samahu	7.8	1.9	2.3	1.3	17	1.8	0.80	0.50	34
Abekuase	5.4	1.8	0.56	0.92	7.5	1.3	0.37	0.53	18
Tebe	4.9	0.79	0.77	1.3	7.6	1.6	0.39	0.25	17
Huniso	2.5	3.9	0.49	0.99	22	0.98	1.3	1.2	33
Pepesa	8.4	3.1	0.73	1.6	33	1.4	0.53	1.0	**51**
T–Cyanide	4.7	6.1	1.4	2.0	29	2.3	1.5	0.69	48
T–Layout	4.7	8.3	0.98	1.7	19	2.2	2.6	1.0	40
T–Brofuyedu	14	4.4	1.1	1.8	10	2.1	0.58	0.45	35
T–Achapime	2.5	3.5	0.73	1.1	**120**	1.4	0.62	0.62	**131**
T–Tamso	**46**	33	4.7	4.0	**69**	21	1.3	1.6	**182**
Minimum	1.6	0.79	0.49	0.53	3.1	0.75	0.15	0.13	9.9
Maximum	46	33	4.7	4.0	400	21	2.6	1.6	427
Average	7.8	4.2	1.2	1.4	61	2.6	0.66	0.57	80
Median	4.7	1.9	0.92	1.3	21	1.8	0.39	0.50	35

Notes: Bold *E^i^_r_* and RI indicates moderate to high risk of heavy metals and/or metalloid.

The RI (range, 9.93–427; mean, 80.4) suggested a low to high risk of heavy metals in the ecological system (plants, soil invertebrates and/or mammalian wildlife) in Mile 7 and Badukrom, respectively. Referring to the classification suggested by Zhu *et al.* [44] (Table 3), soil samples in 10% of the communities could be classified as causing high potential ecological risk to plants, soil invertebrates and/or mammalian wildlife, and another 10% causing considerable potential ecological risk. However soil samples in 5% of the communities could be classified as causing moderate potential ecological risk, while 75% could be classified as causing low potential ecological risk (Table 3 and Table 5). As and Hg, on average, made up 10 and 75% of the RI values, respectively. Overall, the RI of heavy metals in agricultural soils in Tarkwa represented moderate ecological risk. The concentrations of As, Cu, Ni and Pb from the sample sites negatively correlated (*p* ˂ 0.05) with the average distance (km) from the mines (Table 6). The results further suggested that mining activities have played significant roles in the levels, distribution and risk of metals within the study area, especially, the communities closer to the mines.

**Table 6:** Pearson’s correlation matrix of heavy metal concentrations and average distance from the mines.

Metals/Average Distance	Average Distance
average distance	1.0
As	−0.65 **
Cd	−0.38
Co	−0.26
Cr	−0.36
Cu	−0.57 **
Hg	−0.19
Ni	−0.49 *
Pb	−0.53 *
Zn	−0.28

Notes: * Indicates *p* ˂ 0.05; ** Indicates *p* ˂ 0.01.

## 4. Conclusions

The average concentrations of eight metals and a metalloid in agricultural soils in Tarkwa, Ghana decreased in an order of Zn ˃ Cr ˃ Pb ˃ Cu ˃ As ˃ Ni ˃ Co ˃ Hg ˃ Cd. The C_deg_ for Wangarakrom (11), Badukrom (13) and T-Tamso (17) indicated high pollution of toxic metals, especially from As and Hg. The maximum *E^i^_r_* values for As (46) and Hg (400), suggested moderate to very high ecological risk in T-Tamso and Badukrom, respectively. The potential ecological risk indices and potential toxicity response indices of heavy metals and a metalloid indicated low (Mile 7) to high risks (Wangarakrom and Badukrom). Based on the estimates of *C^i^_f_*, C_deg_, *E^i^_r_*, and RI, the investigated soils was within low to high contamination and risk of heavy metals to the ecological system especially plants, soil invertebrates and/or mammalian wildlife. This represented moderate potential ecological risk in the study area and mining activities have played a significant role.

With the rapid increase in mining in Ghana, the local governments should consider the following: (1) increasing investments in environmental pollution monitoring and management, (2) strictly controlling and reducing the sources of heavy metals and metalloids, (3) providing resources to educate the public, to increase awareness about environmental protection since the local people engage in illegal mining activities within the study areas, and (4) continuous screening and monitoring of heavy metals and metalloids in the study area.

## References

[1] Lim H.S., Lee J.S., Chon H.T., Sager M. (2008). Heavy metal contamination and health risk assessment in the vicinity of the abandoned Songcheon Au-Ag mine in Korea. J. Geochem. Explor..

[2] Kumar K.S., Sajwan K.S., Richardson J.P., Kannan K. (2008). Contamination profiles of heavy metals, organochlorine pesticides, polycyclic aromatic hydrocarbons and alkylphenols in sediment and oyster collected from Marsh/Estuarine Savannah GA, USA. Mar. Pollut. Bull..

[3] Wei B., Yang L. (2010). A review of heavy metal contaminations in urban soils, urban road dusts and agricultural soils from China. Microchem. J..

[4] Varol M. (2011). Assessment of heavy metal contamination in sediments of the Tigris River (Turkey) using pollution indices and multivariate statistical techniques. J. Hazard. Mater..

[5] Yaylali-Abanuz G. (2011). Heavy metal contamination of surface soil around Gebze industrial area, Turkey. Microchem. J..

[6] Mireles F., Davila J.I., Pinedo J.L., Reyes E., Speakman R.J., Glascock M.D. (2012). Assessing urban soil pollution in the cities of Zacatecas and Guadalupe, Mexico by instrumental neutron activation analysis. Microchem. J..

[7] Smedley P.L., Kinniburgh D.G. (2002). A review of the source, behavior and distribution of arsenic in natural waters. Appl. Geochem..

[8] Asante K.A., Agusa T., Subramanian A., Ansa–Asare O.D., Biney C.A., Tanabe S. (2007). Contamination status of arsenic and other trace elements in drinking water and residents from Tarkwa, a historic mining township in Ghana. Chemosphere.

[9] Obiri S. (2007). Determination of heavy metals in water from boreholes in Dumasi in the Wassa west district of the western region of the Republic of Ghana. Environ. Monit. Assess..

[10] Akoto O., Bortey–Sam N., Nakayama S., Ikenaka Y., Baidoo E., Yohannes Y.B., Mizukawa H., Ishizuka M. (2014). Distribution of heavy metals in organs of sheep and goat reared in Obuasi: A gold mining town in Ghana. Int. J. Environ. Sci. Toxic..

[11] Bortey-Sam N., Nakayama S.M.M., Ikenaka Y., Akoto O., Yohannes Y.B., Baidoo E., Mizukawa H., Ishizuka M. (2015). Human health risks from metals and metalloid via consumption of food animals near gold mines in Tarkwa, Ghana: Estimation of the daily intakes and target hazard quotients (THQs). Ecotoxicol. Environ. Saf..

[12] Khan S., Cao Q., Zheng Y., Huang Y., Zhu Y. (2008). Health risks of heavy metals in contaminated soils and food crops irrigated with wastewater in Beijing, China. Environ. Pollut..

[13] Nagajyoti P.C., Lee K.D., Sreekanth T.V.M. (2010). Heavy metals, occurrence and toxicity for plants: A review. Environ. Chem. Lett..

[14] Alloway B.J. (2013). Heavy Metals in Soils–Trace Metals and Metalloids in Soils and Their Bioavailability.

[15] Hayford E.K., Amin A., Osae E.K., Kutu J. (2008). Impact of gold mining on soil and some staple foods collected from selected mining communities in and around Tarkwa Prestea Area. West Afr. J. Appl. Ecol..

[16] (2010). Ghana Statistical Service. Population and Housing Census. https://www.google.co.uk/#q=Ghana+Statistical+Service%2C+2010.+Population+and+Housing+census+pp93.

[17] Akabzaa T., Darimani A. (2001). Impact of mining sector investment in Ghana: A study of the Tarkwa mining region. Health Impacts.

[18] Bortey-Sam N., Ikenaka Y., Nakayama S.M.M., Akoto O., Yohannes Y.B., Baidoo E., Mizukawa H., Ishizuka M. (2014). Occurrence, distribution, sources and toxic potential of polycyclic aromatic hydrocarbons (PAHs) in surface soils from the Kumasi Metropolis, Ghana. Sci. Total Environ..

[19] United States Environmental Protection Agency (USEPA) Guidance for Developing Ecological Soil Screening Levels. http://www.epa.gov/ecotox/ecossl/.

[20] United States Environmental Protection Agency (USEPA) (2004). Framework for Inorganic Metals Risk Assessment.

[21] Kabata-Pendias A., Sadurski W. (2004). Trace elements and compounds in soil. In: Elements and Their Compounds in the Environment.

[22] Manta D.S., Angelone M., Bellanca A., Neri R., Sprovieri M. (2002). Heavy metals in urban soils: A case study from the city of Palermo (Sicily), Italy. Sci. Total Environ..

[23] Hani A., Pazira E. (2011). Heavy metals assessment and identification of their sources in agricultural soils of Southern Tehran, Iran. Environ. Monit. Assess..

[24] Kabata-Pendias A., Pendias H. (1992). Trace Elements in Soils and Plants.

[25] Hernandez L., Probst A., Probst J.L., Ulrich E. (2003). Heavy metal distribution in some French forest soils: evidence for atmospheric contamination. Sci. Total Environ..

[26] Gjoka F., Felix-Henningsen P., Wegener H.R., Salillari I., Beqiraj A. (2011). Heavy metals in soils from Tirana (Albania). Environ. Monit. Assess..

[27] Lu A.X., Wang J.H., Qin X.Y., Wang K.Y., Han P., Zhang S.Z. (2012). Multivariate and geostatistical analyses of the spatial distribution and origin of heavy metals in the agricultural soils in Shunyi, Beijing, China. Sci. Total Environ..

[28] Al–Khashman O.A., Shawabkeh R.A. (2006). Metals distribution in soils around the cement factory in southern Jordan. Environ. Pollut..

[29] Chen T., Liu X., Zhu M., Zhao K., Wu J., Xu J., Huang P. (2008). Identification of trace element sources and associated risk assessment in vegetable soils of the urban–rural transitional area of Hangzhou, China. Environ. Pollut..

[30] Grant C., Sheppard S. (2008). Fertilizer impacts on cadmium availability in agricultural soils and crops. Hum. Ecol. Risk Assess..

[31] Suresh G., Sutharsan P., Ramasamy V., Venkatachalapathy R. (2012). Assessment of spatial distribution and potential ecological risk of the heavy metals in relation to granulometric contents of Veeranam lake sediments, India. Ecotoxicol. Environ. Saf..

[32] Amonoo–Neizer E.H., Nyamah D., Bakiamoh S.B. (1995). Mercury and Arsenic Pollution in soil and biological samples around mining towns if Obuasi, Ghana. Water Air Soil Poll..

[33] Streets D.G., Hao J.M., Wu Y., Jiang J.K., Chan M., Tian H.Z., Feng X.B. (2005). Anthropogenic mercury emissions in China. Atmos. Environ..

[34] Manno E., Varrica D., Dongarra G. (2006). Metal distribution in road dust samples collected in an urban area close to a petrochemical plant at Gela, Sicily. Atmos. Environ..

[35] Chen H.M., Zheng C.R., Tu C., Zhu Y.G. (1999). Heavy metal pollution in soils in China: status and countermeasures. Ambio.

[36] Nicholson F., Smith S., Alloway B., Carlton–Smith C., Chambers B. (2003). An inventory of heavy metals inputs to agricultural soils in England and Wales. Sci. Total Environ..

[37] Zhang C.S. (2006). Using multivariate analyses and GIS to identify pollutants and their spatial patterns in urban soils in Galway, Ireland. Environ. Pollut..

[38] Jiao W., Chen W., Chang A.C., Page A.L. (2012). Environmental risks of trace elements associated with long-term phosphate fertilizers applications: a review. Environ. Pollut..

[39] Luo L., Ma Y.B., Zhang S.Z., Wei D.P., Zhu Y.G. (2009). An inventory of trace element inputs to agricultural soils in China. J. Environ. Manag..

[40] Jiang P., Jin S.Y., Hao X.Z., Zhou D.M., Li L.Z., Lv J.L. (2010). Distribution characteristics of heavy metals in feeds, pig manures, soils and vegetables. J. Agro. Environ. Sci..

[41] (2002). Bogoso Gold Limited: Environmental Impact Assessment for Prestea North Project.

[42] BGL, Bogoso Gold Limited (2002). An Overview of Bogoso Gold Limited.

[43] Hakanson L. (1980). An ecological risk index for aquatic pollution control: A sedimentological approach. Water Res..

[44] Zhu W., Bian B., Li L. (2008). Heavy metal contamination of road deposited sediments in a medium size city of China. Environ. Monit. Assess..

[45] Chen T.B., Zheng Y.M., Lei M., Huang Z.C., Wu H.T., Chen H., Fan K.K., Yu K., Wu X., Tian Q.Z. (2005). Assessment of heavy metal pollution in surface soils of urban parks in Beijing, China. Chemosphere.

[46] Loska K., Wiechula D. (2003). Application of principal component analysis for the estimation of source of heavy metal contamination in surface sediments from the Rybnik Reservoir. Chemosphere.

